# Text Messaging to Improve Hypertension Medication Adherence in African Americans From Primary Care and Emergency Department Settings: Results From Two Randomized Feasibility Studies

**DOI:** 10.2196/mhealth.6630

**Published:** 2017-02-01

**Authors:** Lorraine Buis, Lindsey Hirzel, Rachelle M Dawood, Katee L Dawood, Lauren P Nichols, Nancy T Artinian, Loren Schwiebert, Hossein N Yarandi, Dana N Roberson, Melissa A Plegue, LynnMarie C Mango, Phillip D Levy

**Affiliations:** ^1^ Department of Family Medicine University of Michigan Ann Arbor, MI United States; ^2^ College of Nursing Wayne State University Detroit, MI United States; ^3^ Department of Emergency Medicine School of Medicine Wayne State University Detroit, MI United States; ^4^ College of Engineering Wayne State University Detroit, MI United States

**Keywords:** cell phone, text messaging, hypertension, blood pressure, African Americans, medication adherence, telemedicine

## Abstract

**Background:**

Hypertension (HTN) is an important problem in the United States, with an estimated 78 million Americans aged 20 years and older suffering from this condition. Health disparities related to HTN are common in the United States, with African Americans suffering from greater prevalence of the condition than whites, as well as greater severity, earlier onset, and more complications. Medication adherence is an important component of HTN management, but adherence is often poor, and simply forgetting to take medications is often cited as a reason. Mobile health (mHealth) strategies have the potential to be a low-cost and effective method for improving medication adherence that also has broad reach.

**Objective:**

Our goal was to determine the feasibility, acceptability, and preliminary clinical effectiveness of BPMED, an intervention designed to improve medication adherence among African Americans with uncontrolled HTN, through fully automated text messaging support.

**Methods:**

We conducted two parallel, unblinded randomized controlled pilot trials with African-American patients who had uncontrolled HTN, recruited from primary care and emergency department (ED) settings. In each trial, participants were randomized to receive either usual care or the BPMED intervention for one month. Data were collected in-person at baseline and one-month follow-up, assessing the effect on medication adherence, systolic and diastolic blood pressure (SBP and DBP), medication adherence self-efficacy, and participant satisfaction. Data for both randomized controlled pilot trials were analyzed separately and combined.

**Results:**

A total of 58 primary care and 65 ED participants were recruited with retention rates of 91% (53/58) and 88% (57/65), respectively. BPMED participants consistently showed numerically greater, yet nonsignificant, improvements in measures of medication adherence (mean change 0.9, SD 2.0 vs mean change 0.5, SD 1.5, *P*=.26), SBP (mean change –12.6, SD 24.0 vs mean change –11.3, SD 25.5 mm Hg, *P*=.78), and DBP (mean change –4.9, SD 13.1 mm Hg vs mean change –3.3, SD 14.3 mm Hg, *P*=.54). Control and BPMED participants had slight improvements to medication adherence self-efficacy (mean change 0.8, SD 9.8 vs mean change 0.7, SD 7.0) with no significant differences found between groups (*P*=.92). On linear regression analysis, baseline SBP was the only predictor of SBP change; participants with higher SBP at enrollment exhibited significantly greater improvements at one-month follow-up (β=–0.63, *P*<.001). In total, 94% (51/54) of BPMED participants agreed/strongly agreed that they were satisfied with the program, regardless of pilot setting.

**Conclusions:**

Use of text message reminders to improve medication adherence is a feasible and acceptable approach among African Americans with uncontrolled HTN. Although differences in actual medication adherence and blood pressure between BPMED and usual care controls were not significant, patterns of improvement in the BPMED condition suggest that text message medication reminders may have an effect and fully powered investigations with longer-term follow-up are warranted.

**Trial Registration:**

Clinicaltrials.gov NCT01465217; https://clinicaltrials.gov/ct2/show/NCT01465217 (Archived by WebCite at http://www.webcitation.org/6V0tto0lZ).

## Introduction

Hypertension (HTN) is a key risk factor for heart disease and stroke [1], with an estimated 78 million Americans aged 20 years and older suffering from this condition [2]. It is associated with significant health disparities [3], as HTN is more prevalent among non-Hispanic blacks than non-Hispanic whites (42.0% vs 28.8%, respectively [3]), and African Americans suffer from greater disease severity, with earlier onset and more complications than age-matched whites [4].

Adherence to medication regimens is an important component of HTN management [5]; however, only half of all hypertensive patients are considered adherent [6,7]. Forgetting to take medications is one of the most commonly cited reasons for nonadherence [8]. Mobile health (mHealth) strategies, such as text message reminders, could be a low-cost and effective way to improve medication adherence that has broad reach. Cell phone use is widespread, with text messaging even more common. Among American adults, 90% own a cell phone [9] and 81% send text messages [10]. Mobile interventions could be particularly effective among African Americans as studies suggest African-American adults are more likely to own a mobile phone (70% vs 61%) [11] and use it as their primary source of Internet access [11].

The goal of this project was to determine the feasibility and acceptability of BPMED, an automated text messaging system designed to improve medication adherence among African Americans with uncontrolled HTN. We also sought to determine the preliminary effectiveness of this approach compared to usual care controls at one-month follow-up. Our primary outcome measure was medication adherence, with secondary outcome measures of blood pressure (BP) and medication adherence self-efficacy. To account for different ways that African-American patients might interact with the health care system, we conducted two parallel pilot randomized controlled trials (RCTs) with participants recruited from primary care and emergency department (ED) settings.

## Methods

We developed BPMED, an automated text message medication reminder system, to assist African Americans with uncontrolled HTN in remembering to take their HTN medications. BPMED’s development and study protocol are described in detail elsewhere [12]; however, key elements are summarized subsequently. The Wayne State University Institutional Review Board (#0410810B3E) approved this study.

### Study Design

We simultaneously conducted two unblinded parallel pilot RCTs with participants recruited from primary care and ED settings. Within each pilot RCT, block randomization, with blocks of 10 generated by the study biostatistician, was used to allocate participants equally to receive usual care or BPMED for one month. Blinded group assignments were concealed in an unmarked sealed envelope, which was included with the consent and enrollment packet, and were only opened once a participant was consented. Because many ED participants were not currently taking antihypertensive medications, all ED trial participants were given a 35-day supply of medication.

### Recruitment

Primary care participants were recruited from primary care clinics in Detroit and Southfield, MI. Many were affiliated with MetroNet, a practice-based research network in Southeast Michigan. Primary care participants were recruited via provider referral, signs posted in clinic exam rooms, and targeted recruitment letters sent to potentially eligible participants. The ED participants were recruited from a large, urban ED in Detroit, MI, through real-time monitoring of the ED tracking board by research assistants. For the ED pilot RCT, all recruitment, screening, and enrollment were conducted on site and typically occurred immediately after ED discharge. All participants received US $25 cash for completing each data collection visit (total possible participant incentive=US $50).

### Eligibility Screening and Consent

All potential participants were screened for eligibility, and those eligible were consented, enrolled, and randomized by research staff, followed by baseline data collection.

#### Inclusion Criteria

Potential participants were required to be African American, aged 18 years or older, have a diagnosis of HTN based on *International Classification of Diseases, Ninth Revision* (*ICD-9*) codes documented in the medical record, have a cell phone with text messaging, and speak English. Additionally, primary care participants were required to have uncontrolled HTN documented in their medical record on two successive clinic visits (clinic systolic blood pressure [SBP] >140 mm Hg and diastolic blood pressure [DBP] >90 mm Hg or SBP >130 mm Hg and DBP >80 mm Hg for those with diabetes or kidney disease) and be taking at least one antihypertensive medication. For the ED cohort, presence of an elevated BP (SBP >140 mm Hg) on successive measurements obtained at least one hour apart was required. All BPs were obtained using automated brachial cuff devices, with the participant seated or supine, and the measurement arm supported at the midsternal level.

#### Exclusion Criteria

Potential participants were excluded if they self-reported any of the following: strict adherence to antihypertensive medication regimens, undergoing hemodialysis, plans to move more than 50 miles away from the recruitment site or to terminate cell phone contract within the next three months, compliance risk as identified by a score ≥2 on the CAGE Questionnaire Adapted to Include Drugs (CAGE-AID) for substance/alcohol abuse [13], and/or any other major health problem that would make follow-up difficult. Participants with a documented diagnosis of resistant HTN were also excluded.

### BPMED

BPMED is an automated text message system that sends daily medication reminders to users at individually customized times. BPMED also sends two educational messages per week, with content based on HTN management recommendations from the American Heart Association. Topics include smoking cessation, dietary sodium reduction, physical activity, stress, nutrition, weight reduction, and alcohol consumption. BPMED closely aligns with a Health Belief Model [14] framework of behavior change, with medication reminders serving as cues to action. Additional detail on BPMED development has been previously described [12]. Participants who self-reported at baseline that text messaging was not included in their cell phone plan were reimbursed at follow-up US $0.20 per text message sent/received in the study.

### Procedures

#### Measures

Participant data were collected in-person at baseline and at one-month follow-up. Primary care participant data were collected primarily on the university campus where the research was conducted, whereas ED participant data were collected in the ED or another building on campus. Participants completed self-reported assessments either in paper format or electronically via study-furnished laptops. The primary outcome measure was medication adherence as quantified by the Morisky Medication Adherence Scale (MMAS) [15-17]. The MMAS is a self-reported eight-item instrument (total score range 0-8). Participants with scores less than six points are considered to have low medication adherence. Pill counts were obtained as a second measure of medication adherence; however, they were not analyzed due a high degree of missing data. Secondary outcome measures included BP and medication adherence self-efficacy, as well as participant satisfaction. For group comparisons, BP was treated as a continuous variable and absolute differences at one-month follow-up, as well as changes over time, were included. Medication adherence self-efficacy was measured using 21 items from the Medication Adherence Self-Efficacy Scale (MASES) [18], a tool that captures self-efficacy for situational medication adherence. Relevant to our study design, MASES was developed and validated in African-American cohorts.

#### Statistical Analysis

Descriptive statistics for participant characteristics, including demographics, cell phone use, medication adherence, BP, medication adherence self-efficacy, and perceptions of the BPMED intervention were compiled. Missing data from the MMAS was imputed by assigning a zero value for the missing item, which indicated medication nonadherence, so long as no more than two of the eight MMAS items were missing. If more than two items were missing from the scale, the total MMAS score was not computed, and the data were considered missing. Missing data from the MASES was handled through mean imputation of answered items, so long as no more than three of the 21 items were missing. If more than three items were missing, the overall MASES score was not computed and the data were considered missing.

Demographics, baseline medication adherence, BP, and medication adherence self-efficacy, and changes in these measurements from baseline to follow-up, were compared between treatment arms, as well as between primary care and ED settings, using independent samples *t* tests for continuous data and Pearson chi-square for categorical data. We conducted analyses on each of the two pilot RCTs independently and pooled. In the pooled analysis, linear regression was conducted on primary outcome (medication adherence) and secondary outcome measures (SBP, DBP, and medication adherence self-efficacy), including indicators for study setting (primary care vs ED), treatment arm (usual care vs BPMED), baseline SBP, and additional variables that were significantly different between the two pilot studies. Interactions between pilot setting with treatment arm and baseline SBP were also investigated to assess consistent effects on outcomes between studies. All analyses were conducted using Stata 10.0.

## Results

### Recruitment

**Figure 1:**
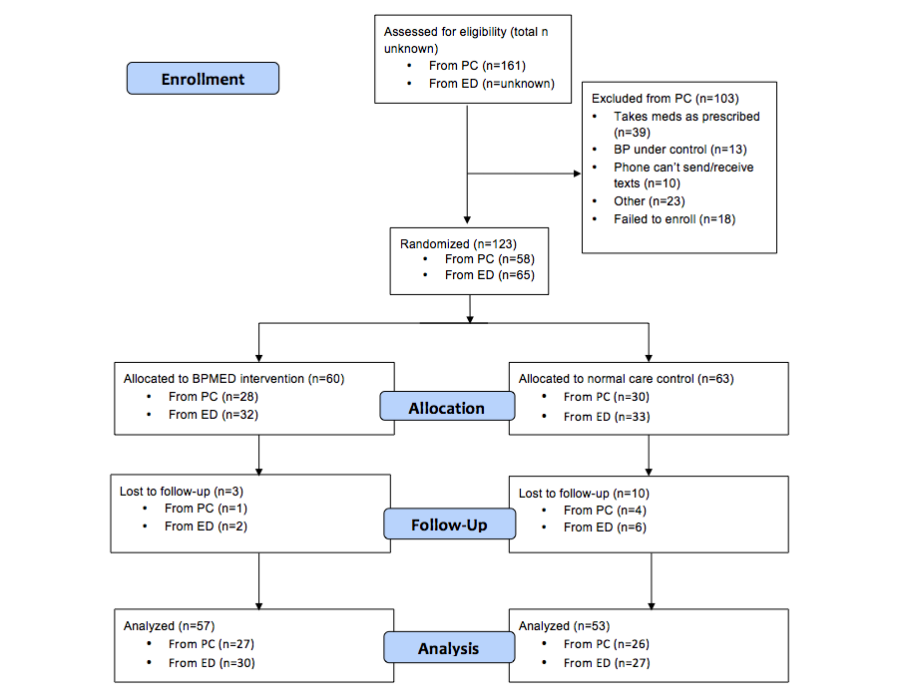
Participant flow through BPMED trials.

We recruited 123 participants for the two pilot RCTs (n=58 for primary care and n=65 for ED) between 2012 and 2014. See Figure 1 for participant flow. The sample was primarily female (55.4%, 67/121) with a mean age of 49.0 (SD 8.3) years. The majority had a cell phone plan that included text messaging (90.8%, 109/120) and most (65.0%, 78/120) reported daily text message use. See Table 1 for participant characteristics.

**Table 1:** Participant demographics.

Characteristic		Primary care	Emergency department	Total
Age (years),^a^ mean (SD)		52.2 (7.6)	46.3 (8.0)	49.0 (8.3)
**Gender,^a^** **n (%)**		n=56	n=65	n=121
	Female	37 (66)	30 (46)	67 (55.4)
	Male	19 (34)	35 (54)	54 (44.6)
**Highest level of education, n (%)**		n=55^b^	n=65	n=120
	Some high school	7 (13)	15 (23)	22 (18.3)
	High school diploma or GED	16 (29)	21 (32)	37 (30.8)
	Some college	16 (29)	22 (34)	38 (31.7)
	Associates degree	7 (13)	5 (8)	12 (10.0)
	Bachelor’s degree or higher	9 (16)	2 (3)	11 (9.2)
**Marital status, n (%)**		n=56^c^	n=65	n=121^c^
	Single, never married	27 (48)	42 (65)	69 (57.0)
	Married	7 (13)	9 (14)	16 (13.2)
	Separated/Divorced	16 (29)	12 (18)	28 (23.1)
	Widowed	6 (11)	2 (3)	8 (6.6)
**Annual household income (US $), n (%)**		n=55^c^	n=65	n=120
	<10,000	25 (45)	34 (52)	59 (49.2)
	10,000-19,999	15 (27)	7 (11)	22 (18.3)
	≥20,000	15 (27)	24 (37)	39 (32.5)
**Employment status,^a^** **n (%)**		n=56	n=65	n=121^c^
	Work part time	5 (9)	12 (18)	17 (14.0)
	Work full time	12 (21)	23 (35)	35 (28.9)
	Retired	4 (7)	1 (2)	5 (4.1)
	On disability	21 (38)	2 (3)	23 (19.0)
	Laid off/unemployed	14 (25)	27 (42)	41 (33.9)
**Cell phone plan is prepaid (phone cards), n (%)**		n=55	n=65	n=120^c^
	Yes	6 (11)	5 (8)	11 (9.2)
	No	47 (85)	60 (92)	107 (89.2)
	Don’t know	2 (4)	0 (0)	2 (1.7)
**Length of current cell phone plan ownership, n (%)**		n=53	n=64	n=117
	≤6 months	9 (17)	8 (13)	17 (14.5)
	7-12 months	9 (17)	4 (6)	13 (11.1)
	>1 year	35 (66)	52 (81)	87 (74.4)
**Frequency of text message use, n (%)**		n=55^c^	n=65	n=120
	Never	2 (4)	1 (2)	3 (2.5)
	A few times per month	7 (13)	12 (18)	19 (15.8)
	A few times per week	11 (20)	9 (14)	20 (16.7)
	Daily	35 (64)	43 (66)	78 (65.0)

^a^ Significant difference between primary care and ED (*P*<.05).

^b^ Significant difference between arms.

^c^ Sum total does not equal 100% due to rounding error.

### Differences Between Primary Care and Emergency Department Samples

On average, ED participants were younger (mean 46.3, SD 8.0 years vs mean 52.2, SD 7.6 years; *P*<.001), less likely to be female (46%, 30/65 vs 66%, 37/56; *P*=.03), and more likely to be employed (employed part/full time: 54%, 35/65 vs 30%, 17/56; *P*<.001). Although patients recruited from both settings had suboptimal BP at baseline, ED participants had significantly higher SBP (mean 165.2, SD 19.2 mm Hg vs mean 136.2, SD 22.2 mm Hg; *P*<.001) and DBP (mean 97.8, SD 12.7 mm Hg vs mean 89.4, SD 11.2 mm Hg; *P*<.001). Additionally, primary care participants had significantly lower medication adherence self-efficacy than ED participants (MASES: mean 46.7, SD 10.9 vs mean 52.7, SD 8.5; *P*=.001).

### Effects of BPMED

A majority of primary care (91%, 53/58) and ED (88%, 57/65) participants completed the one-month follow-up. Although results were analyzed separately for each pilot study, intervention effects were consistent between the two settings, as well as with combined results; therefore, pooled analyses are discussed. Summary data (Table 2) are presented as individual pilot and combined primary and secondary outcome means.

**Table 2:** Summary of mean primary and secondary outcome measures by study setting and combined.

Measure		Primary care		Emergency department		Pooled	
		Control	BPMED	Control	BPMED	Control	BPMED
**MMAS (points)**							
	Baseline	4.4	4.6	4.8	5.0	4.6	4.8
	Follow-up	4.7	5.2	5.7	6.3	5.2	5.8
	Change	0.3	0.6	0.7	1.1	0.5	0.9
**SBP (mm Hg)**							
	Baseline	135.4	137.0	164.6	165.9	151.0	152.7
	Follow-up	133.4	133.1	147.1	146.5	140.4	140.2
	Change	–3.1	–4.6	–18.9	–19.5	–11.3	–12.6
**DBP (mm Hg)**							
	Baseline	88.5	90.3	97.8	97.8	93.5	94.4
	Follow-up	86.8	87.5	93.9	92.5	90.4	90.2
	Change	–1.7	–3.0	–4.7	–6.5	–3.3	–4.9
**MASES (points)**							
	Baseline	46.8	46.7	51.3	54.1	49.1	51.1
	Follow-up	48.1	49.5	52.3	54.0	50.3	52.0
	Change	0.2	1.6	1.4	–0.0	0.8	0.7

#### Medication Adherence, Blood Pressure, and Medication Adherence Self-Efficacy

At follow-up, BPMED participants experienced greater, yet nonsignificant, mean improvements on the MMAS scale compared to usual care (mean change 0.9, SD 2.0 vs mean change 0.5, SD 1.5; *P*=.26). Both control and BPMED participants had improved SBP (mean 140.4, SD 22.0 mm Hg and mean 140.2, SD 21.6 mm Hg, respectively) and DBP (mean 90.4, SD 11.8 mm Hg and mean 90.2, SD 13.6 mm Hg, respectively) at follow-up, but BPMED participants experienced greater, yet nonsignificant, mean improvements in BP compared to usual care (SBP: mean change –12.6, SD 24.0 and mean change –11.3, SD 25.5 mm Hg, *P*=.78; DBP: mean change –4.9, SD 13.1 mm Hg and mean change –3.3, SD 14.3 mm Hg, *P*=.54). However, negligible improvements in medication adherence self-efficacy were noted (MASES: mean change 0.8, SD 9.8 and mean change 0.7, SD 7.0, respectively) with no significant differences found between groups (*P*=.92). Outcomes were similar when analyzed separately by pilot study group.

#### Predictors of Change in Medication Adherence, Blood Pressure, and Medication Adherence Self-Efficacy

No significant predictors of change in medication adherence, DBP, or medication adherence self-efficacy were found in linear regression (Table 3). Baseline SBP was found to be a significant predictor of overall change in SBP (β=–0.63, *P*<.001), with higher baseline SBPs associated with greater change. Because ED participants had higher mean baseline SBP, we tested the interaction between study setting and baseline SBP. This interaction was significant and in the same direction for both primary care and ED sites (with different magnitudes), suggesting this effect was not solely due to the presence of ED participants (β=–0.35, *P*=.01 and β=–0.90, *P*<.001, respectively). To ease interpretation of setting effects, the interaction was not retained in the model (see Table 3). No other interaction terms were significant for any of the estimated models; hence, they were not included in the final analyses.

**Table 3:** Regression analyses of treatment arm and pilot study on change in primary and secondary outcome measures.

Outcome variable and independent variable		β (95% CI)	*P* value
**Change medication adherence^a^**			
	Treatment	–0.42 (–1.19, 0.35)	.28
	Pilot setting	0.41 (–0.64, 1.45)	.44
**Change SBP^a^**			
	Treatment	2.68 (–5.73, 11.10)	.53
	Pilot setting	–0.16 (–11.79, 11.47)	.98
**Change DBP^a^**			
	Treatment	2.71 (–2.93, 8.35)	.34
	Pilot setting	2.12 (–5.67, 9.91)	.59
**Change medication adherence self-efficacy^a^**			
	Treatment	–1.40 (–4.46, 1.66)	.37
	Pilot setting	1.89 (–2.34, 6.11)	.38

^a^ Controlling for baseline SBP, age, gender, employment, and baseline medication self-efficacy.

#### Participant Perceptions

The BPMED participants were overwhelmingly satisfied with the program with no significant differences in satisfaction measures between primary care and ED settings. The vast majority agreed/strongly agreed that BPMED was easy to use (98%, 52/53), were satisfied with BPMED (94%, 51/54), would recommend BPMED to others (94%, 51/54), agreed that BPMED helped them remember to take their medications (89%, 48/54), and believed that BPMED benefited their overall health (87%, 47/54). Overall, most (85%, 46/54) participants agreed/strongly agreed that they would like to keep using BPMED; however, this desire was more common among ED vs primary care participants (97%, 29/30 vs 71%, 17/24, respectively).

## Discussion

We sought to document the feasibility, acceptability, and preliminary efficacy of text messages for antihypertensive medication reminders. The BPMED participants were found to be very satisfied with and enthusiastic about the program. This finding is consistent with previous text message medication adherence studies for chronic disease that found moderate to high levels of participant satisfaction [19,20] and general comfort with the technology and message content [21]. Demand for such technology is growing with the existence of a large number of apps (n=193) and websites to send medication reminders and log adherence [22,23]. In 2014, 55% of these medication support apps were tailored toward HTN self-monitoring [22,23].

With expanding interest in mHealth interventions, and increasing emphasis on prevention, there have been calls for large-scale RCTs aimed specifically at chronic conditions such as HTN [24]. Our data support this, showing greater numerical improvements in medication adherence at one-month follow-up among individuals randomized to BPMED. Although this was not statistically significant, our sample was underpowered to detect modest differences, and intervention effects may be less pronounced over a short-term follow-up period. Existing literature related to text messaging for medication adherence in chronic disease is mixed [24], but a recent meta-analysis of 16 RCTs found a positive overall effect, suggesting a 17.8% increase in medication adherence rates (from an assumed 50% baseline adherence rate to 67.8%, OR 2.11, 95% CI 1.52-2.93, *P*<.001) over a mean follow-up period of 12 weeks (range 4-48 weeks) [20]. Thus, we are encouraged to continue research into the potential benefits of text messaging on medication adherence in this population, and are currently conducting a well-powered RCT of mHealth support to improve BP in a cohort similar to our ED trial, with one-year follow-up (NCT02955537). Our finding of near universal support for continued use of BPMED among ED participants lends further credence to this approach.

Regarding medication adherence self-efficacy (a secondary outcome of interest), only minor, nonsignificant improvements were seen among control and BPMED participants. This finding was unexpected as there is an established connection between medication adherence self-efficacy and medication adherence [25,26]. Our small sample size may have been insufficient to reveal differences in medication adherence self-efficacy. Individuals who received BPMED had numerically greater, yet nonsignificant, improvements in SBP and DBP at follow-up. Although there are few studies of text messaging to improve medication adherence targeting patients with uncontrolled HTN, or evaluating direct effects on BP, this trend is consistent with previous work that has established greater reductions in cholesterol and SBP at six months for patients with heart disease who received a mHealth program compared to controls [27].

These pilots were intended to demonstrate feasibility and acceptability of our approach. As such, we had relatively small sample sizes that contributed to our lack of statistically significant effects of BPMED on our primary and secondary outcomes. As noted, these limitations are consistent with other work focused on text message reminders for medication adherence in chronic disease. This highlights the need for larger RCTs of text message medication reminders with longer duration follow-up, a need that has been noted in the literature [20]. The measurement of medication adherence utilizing the MMAS was also a limitation. Although its use is well documented [20], the MMAS is a self-reported measure, not an objective assessment, which can suffer from overestimation of medication adherence [28-30]. Future work should incorporate better measures, including instrumented pill bottles/caps [31] and/or biomarkers that may provide better approximations of adherence [32,33]. Because control participants knew they were in a study about medication adherence, a Hawthorne effect may have contributed to the lack of statistically significant between group differences. This may equal out over longer follow-up periods, particularly with less overt measures of medication adherence. Ultimately, BPMED uses a single component approach to improve medication adherence, perhaps the most important limiting factor. Previous work suggests that multicomponent interventions are more effective at improving medication adherence [23]. Interventions that include bidirectional texting, allowing participants to respond with adherence information, have been found to be significantly more effective than those employing unidirectional text message reminders (relative risk 1.0 vs 1.2, respectively) [34,35]. Moreover, technology-augmented mechanisms, such as instrumented pill bottles, have shown to increase adherence more than text messaging alone [36]. Other similar interventions that utilize pill top monitors and triggered text message reminders when doses are late or skipped found that 87.3% of intervention participants reached 95% or more on-time adherence compared to 61.8% among controls [31]. Future work should take a more robust approach to improving medication adherence, as single component interventions may not be as effective.

Our results demonstrate that text message reminders to improve medication adherence among African Americans with uncontrolled HTN are feasible and acceptable. Our data support the need for more robust trials of mHealth in patients with uncontrolled chronic HTN that are fully powered with longer follow-up periods, more rigorous measures of medication adherence, and multimodal interventions.

## Abbreviations

BP: blood pressure
DBP: diastolic blood pressure
ED: emergency department
HTN: hypertension
MASES: Medication Adherence Self-Efficacy Scale
mHealth: mobile health
MMAS: Morisky Medication Adherence Scale
RCT: randomized controlled trial
SBP: systolic blood pressure
